# Supplementary material for: Foot-and-Mouth Disease Virus-like Particles Produced in E. coli as Potential Antigens for a Novel Vaccine
Source: Vet Sci. 2025 Jun 2;12(6):539. doi: 10.3390/vetsci12060539 (PMC12197693; doi:10.3390/vetsci12060539)
Supplement: Supplementary file 1 [file vetsci-12-00539-s001.zip › Table S1. Primers used for PCR amplification and gene assembly..pdf]

Vector	Virs	Name	Primer Sequence (5ʹ - 3ʹ)
MCS1	A YC	AYCP1-2A-MCS1-F	CTTTAAGAAGGAGATATACCATGGGGGC CGGGCAATCCAG
		AYCP1-2A-MCS1-R	GTGGGGCACCACTTGAATTCCCGGGGTT GGACTCAACGTC
		3C-MCS1-F	ACAGCCAGGATCCGAATTCAAGTGGTGC CCCACCGACCGA
		3C-MCS1-R	TTAAGCATTATGCGGCCGCTCACTCGTG GTGTGGTTCAGGGT
	O PA2	OPA2P1-2A-MCS1-F	TAATAAGGAGATATACCATGGGCGCCGG GCAATCCAGCCC
		OPA2P1-2A-MCS1-R	GTGGGGCACCACTTGAATTCCCAGGGTT GGACTCAACGTC
		3C-MCS1-F	ACAGCCAGGATCCGAATTCAAGTGGTGC CCCACCGACCGA
		3C-MCS1-R	TTAAGCATTATGCGGCCGCTCACTCGTG GTGTGGTTCAGGGT
	Asia1 Shamir	Asia1ShamirP1-2A-MCS1-F	CTTTAAGAAGGAGATATACCATGGGAGC CGGTCAATCCAG
		Asia1ShamirP1-2A-MCS1-R	GTGGGGCACCACTTGAATTCCCAGGGTT GGACTCAACGTC
		3C-MCS1-F	ACAGCCAGGATCCGAATTCAAGTGGTGC CCCACCGACCGA
		3C-MCS1-R	TTAAGCATTATGCGGCCGCTCACTCGTG GTGTGGTTCAGGGT
MCS2	A YC	AYCP1-2A-MCS2-F	TATAAGAAGGAGATATACATATGGGGGCC GGGCAATCCAG
		AYCP1-2A-MCS2-R	GTGGGGCACCACTTGATATCCCGGGGTT GGACTCAACGTC
		3C-MCS2-F	GCAGATCTCAATTGGATATCAAGTGGTG CCCCACCGACCG
		3C-MCS2-R	GTTTCTTTACCAGACTCGAGTCACTCGT GGTGTGGTTCAG
	O PA2	OPA2P1-2A-MCS2-F	AAGAAGGAGATATACATATGGGCGCCGG GCAATCCAGCCC
		OPA2P1-2A-MCS2-R	GTGGGGCACCACTTGATATCCCAGGGTT GGACTCAACGTC
		3C-MCS2-F	GCAGATCTCAATTGGATATCAAGTGGTG CCCCACCGACCG
		3C-MCS2-R	GTTTCTTTACCAGACTCGAGTCACTCGT GGTGTGGTTCAG

Asia1 Shamir	Asia1ShamirP1-2A-MCS1-F	TATAAGAAGGAGATATACATATGGGAGCCGGTCAATCCAG
	Asia1ShamirP1-2A-MCS1-R	GTGGGGCACCACTTGATATCCCAGGGTTGGACTCAACGTC
	3C-MCS2-F	GCAGATCTCAATTGGATATCAAGTGGTGCCCCACCGACCG
	3C-MCS2-R	GTTTCTTTACCAGACTCGAGTCACTCGTGGTGTGGTTCAG
