# Supplementary figures and images for: Foot-and-Mouth Disease Virus-like Particles Produced in E. coli as Potential Antigens for a Novel Vaccine
Source: Vet Sci. 2025 Jun 2;12(6):539. doi: 10.3390/vetsci12060539 (PMC12197693; doi:10.3390/vetsci12060539)

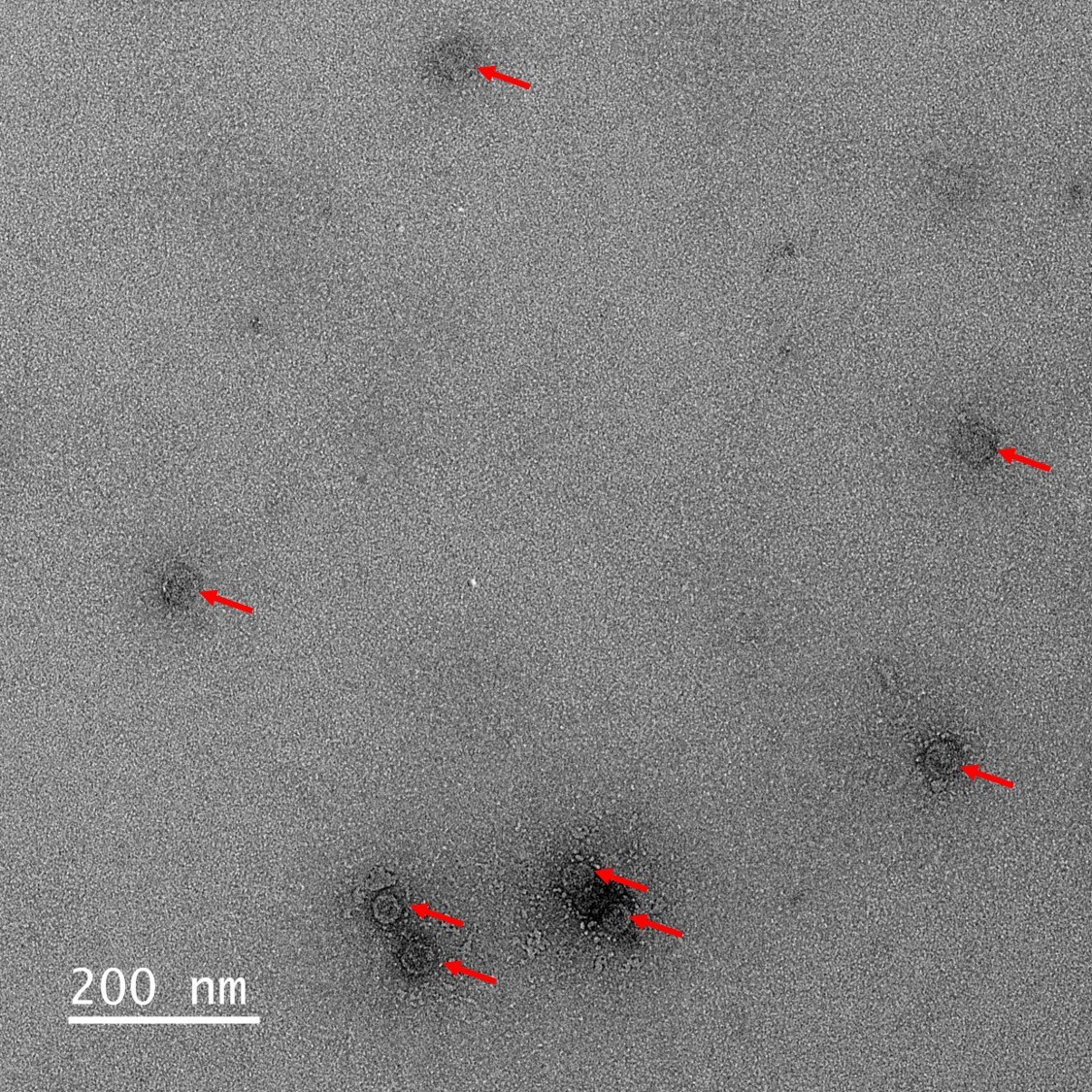

Supplement: Supplementary file 1 [file vetsci-12-00539-s001.zip › Fig. S1. Transmission electron microscopy analysis of sucrose-purified VLPs.jpg]
